# Food allergies in school: design and evaluation of a teacher-oriented training action

**DOI:** 10.1186/s13052-014-0100-8

**Published:** 2014-12-04

**Authors:** Licia Ravarotto, Giulia Mascarello, Anna Pinto, Maria Rita Schiavo, Marina Bagni, Lucia Decastelli

**Affiliations:** Health Awareness and Communication Department, Istituto Zooprofilattico Sperimentale delle Venezie, Viale dell’Università 10, 35020 Legnaro, Padova Italia; Dipartimento di ricerca biotecnologica e diagnostica specialistica, Istituto Zooprofilattico Sperimentale della Sicilia, Via G. Marinuzzi 3, 90129 Palermo, Italia; Dipartimento della sanità pubblica veterinaria, della sicurezza alimentare e degli organi collegiali per la tutela della salute, Ministero della salute, Viale Giorgio Ribotta 5, 00144 Roma, Italia; Controllo Alimenti e Igiene delle Produzioni, Istituto Zooprofilattico Sperimentale Piemonte, Liguria e Valle d’Aosta, via Bologna 148, 10154 Torino, Italia

**Keywords:** Food allergies, Teacher-oriented training, Focus groups, Communication actions

## Abstract

**Background:**

Food allergies are perceived as a significant problem in school environments; as a result, a teacher’s ability to recognise and deal with allergic reactions is of fundamental importance to protect children’s health. This paper includes the results of a study conducted for the purposes of designing, implementing and monitoring a specific set of teacher-oriented communication actions.

**Methods:**

The study involved designing, implementing and assessing five workshops. These workshops were designed on the basis of the analysis of perceptions and information needs investigated by three focus groups (25 teachers). The level of the teachers’ knowledge and appreciation of the workshops was evaluated by using two structured questionnaires (n = 158).

**Results:**

The teachers feel that they are insufficiently informed about food allergies; this knowledge gap is confirmed by an analysis of their knowledge before participating in the workshops. According to the teachers, the information which would be most useful to them has to do with the practical management of allergies in school. They feel that there is a lack of a professional contact person for precise and reliable information on health issues. The workshops seem to be appreciated as an information method. In addition, there appears to be a need to involve all children in awareness raising activities and education projects on this subject.

**Conclusions:**

There is an urgent need for training actions on food allergies in Italian schools, in particular the communication of practical information regarding the management of allergies and emergencies. More communication between the medical and school staff is, in particular, advisable.

## Background

Food allergies are a serious public health issue, with an estimated incidence ranging from 3% to 6% of the general population [1]; they also have significant impact in terms of quality of life for allergy patients [2,3]. Several studies have underscored the increase in these pathologies [1,4-6]; moreover, recent data show that the concern regarding the possibility of an allergic reaction has significantly risen in Europe between 2005 and 2010. More specifically, Italian citizens appear to be the most concerned on this issue [7]. Children are more affected by food allergies than adults [8], and school is definitely a setting where children spend a lot of time and where they come into contact with various kinds of food.

Some studies have shown that nearly one fifth of children suffering from food allergies have experienced at least one allergy reaction in school [9,10]. Moreover, food allergies are one of the most frequent causes of anaphylaxis [11-13] and – as such – they may also affect children who have not been previously diagnosed with any allergy.

Given that the symptoms of an allergy reaction usually appear very rapidly, namely between 5 and 30 minutes following exposure to the allergen [2], it is of fundamental importance for teachers to promptly recognise and deal with such events. It is true that most allergic reactions do not result in anaphylactic shock, however some studies have shown that nearly one third of anaphylactic reactions with a fatal outcome occurring in school are mainly associated with a delayed response [14,15]. As a matter of fact, it appears that teachers are not always trained to deal with this kind of situation [16,17].

Although several studies and communication actions have been targeted to parents of allergic children [18-21], less attention is given to specifically targeting teachers, even though a number of studies have highlighted the need to implement training actions for this professional group and for the school staff in general [16,22-24]. Training is a prerequisite not only in order to make sure that the school staff is more attentive to this issue, but also to help teachers, parents and children reduce the feeling of anxiety which is brought on by this pathology [3]. Food allergies, indeed, can have a substantial impact on the quality of life of the child and the whole family [25]. This is because they limit the range of potential daily activities in addition to having a psychological effect due to the feelings of anxiety and depression that they tend to cause [26-29].

In 2005, the Ministry of Education, in collaboration with the Ministry of Education, University Studies and Research, issued recommendations containing the “Guidelines for the definition of actions to assist students that require drugs during school hours in order to protect their rights to education, health and well being within the school environment”. Each regional council, at its own discretion, may apply the specifications of the Ministry through a decree; this must be immediately followed by a memorandum of agreement between the Region and the regional school office which will regulate the entire procedure for administering drugs during school hours.

In Italy few actions have yet been implemented for the purposes of raising awareness and educating school staff about food allergies [30]. This paper includes the results of an intervention study which was developed in order to involve and inform teachers who mainly work in primary school about the risks associated with food allergies.

### Purpose

The study had three main goals:

- studying the teachers’ perception, experience and information needs, in order to design a suitable communication action in line with the requirements of the target group;
- designing, implementing and monitoring a specific set of teacher-oriented communication actions;
- giving teachers access to educational materials aimed at raising awareness among them and among pupils with regard to food allergies.

## Methods

### Procedure

The research work was divided into three phases. Phase one (exploration) consisted in analysing the teachers’ perceptions, knowledge and information needs in respect of the topic. Phase two (communication) involved five workshops open to teachers and aimed at providing them targeted scientific information. These seminars were an opportunity for a preview of the publication “Il teatro della salute” [The Theatre of Health] [31] which contained some educational materials for involving primary school children in the issue at hand. Phase three (assessment) consisted, on the one hand, in analysing and comparing the teachers’ level of knowledge before and after taking part in the workshop; on the other side it was aimed at monitoring the participants’ level of appreciation with regard to the workshop.

### Phase 1 - exploration: focus groups

During the course of phase 1, data was collected through three focus groups which investigated: interest in the topic on the part the persons involved, events experienced in the school setting in relation to the issue and teachers’ information needs.

The focus group method has made it possible to explore the teachers’ perspective with regard to food allergies, in order to construct an ad-hoc communication strategy for this target. The focus groups were conducted by using the semi-structured interview format. Ten open-ended questions were drafted with a view to highlighting the teachers’ perspective in relation to the established objectives (Table 1). The outline of the focus group was based on the experience of the research team and on existing literature [16,18,19,22,2].

**Table 1:** **Outline of questions for the focus group by domain**

**Domain**	**Questions**
Definition of food allergies	How would you define food allergies?
Perception of importance of food allergies in school	Is it an issue of which teachers are aware? How important is the issue of allergies in school?
Experience in a school environment	Have you ever met students with these problems during your career as a teacher? What problems did you encounter when managing an allergic child in school?
Perception of the level of personal and general knowledge of teachers on this topic	What do you believe is the general level of preparation of teachers? And personally how knowledgeable would you consider yourself to be?
Knowledge and participation in communication and informational initiatives	Have you participated in projects on these topics within school? Initiatives within the territory? Personal initiatives?
Knowledge	Which topics have you studied in depth during courses? (food allergens, symptoms and reactions, management of allergic episodes)
Informational sources of teachers	If you have doubts or need information on these topics, who do you contact? How do you find the answers to your questions?
Informational needs of teachers	Which topics would you like to study in depth? What type of knowledge would you like to receive on this topic?
Communication proposals	How would you like to receive information on this topic?
School and family	What is the relationship between teachers and parents with allergic children?

The focus groups took place between February and March 2012 in three primary schools, in Rome, Turin and Palermo respectively. 25 teachers participated in this phase (10 teachers in Palermo, 10 in Rome and 5 in Turin) and all of them were female, aged between 29 and 63 years.

The teachers participated voluntarily in the focus groups meetings. They were recruited by the headmasters of the primary schools involved who collected the availability of one or two teachers per class.

Each of the sessions lasted 90 minutes and the focus group meetings were all conducted by the same moderator and by the same assistant moderator who took field notes. The sessions were audio-recorded and coded using qualitative analysis techniques. The recordings were manually analysed independently by two codifiers without using any software.

More specifically, the data were analyzed starting from the formulation of a system of open categories [32], which has allowed us to classify thematic categories that were then discussed and shared. This type of coding is part of Grounded Theory, whose strong point is the circularity of the process characterized by the lack of interruption between collection and analysis of data, with a continuous reflection on the research process [33,34].

### Phase 2 - communication: information workshops

The communication phase consisted of five workshops on food allergies. The workshops took place in the cities of Turin, Palermo, Rome, Trento and Genoa; they lasted two hours each. The meetings took place in the assembly hall of five primary schools, one for each selected province, and there were 197 teachers involved.

A majority of the teachers involved were women (95.5%) of an age between 46 and 55 years (43.5%). All the teachers of the affected schools were invited to participate in the workshop by means of informational materials that were distributed in collaboration with school executives. The teachers participated on a voluntary basis and all requests for participation were accepted. The teachers who had participated in the focus group of Phase 1 were also invited to participate in the workshops organized in their city. Recruitment of participants on a voluntary basis was deemed the most suitable procedure for the project.

In fact, given the characteristics of the utilized tool (workshop), it was concluded that participation would be incentivized by leveraging the teachers’ motivation to develop knowledge within a topic that is not directly subject to educational activities but which is essential for the correct management of the organizational wellbeing of the school environment and which is ascribable to individual responsibility.

These workshops included a discussion between teachers and experts in the sector: a doctor (allergist and/or paediatrician, operating in the area where the workshop being organised), a veterinarian and an expert in scientific communication. The contents of the meetings were planned according to specific knowledge requirements of the teachers, in accordance with what emerged during the focus group meetings. In order to avoid any discrepancy in passing on the scientific information due to the involvement of various doctors, the key contents were agreed in detail beforehand, as were the communication methods (a 40-minute presentation with the support of slides in power point format). At the end of each expert report, some space was left for discussion, thereby allowing for certain doubts to be clarified.

The workshops were dedicated to the following subject matters:

- what kind of food contains allergens;
- the difference between allergy and intolerance;
- what are the symptoms and reactions which can be traced back to an allergic event;
- an introduction to first aid;
- the information/training tools regarding allergens available to citizens (allergologists networks, institutional sources);
- the regulations in force to protect consumers.

Moreover, the seminars were an opportunity to distribute, as educational materials, the editorial product “Il teatro della salute” [The Theatre of Health], the latter was generated from a project that was simultaneously conducted with the current research work by the ’Italian Ministry for Health, containing two theatre performances revolving around the issue of food allergies and intolerances [31]. These theatre plays were provided to teachersat the end of each session in order to maintain their attention and interest in the topic.

### Phase 3: workshop assessment

The workshops were assessed through the analysis of two structured questionnaires which were administered to the teachers respectively at the beginning (ex-ante questionnaire) and at the end (ex-post questionnaire) of the workshop.

The questionnaires were delivered to 197 teachers; however, only 158 of them filled them out in their entirety. For purposes of the workshop assessment, therefore, only the 158 who filled out both questionnaires in their entirety were considered.

The ex-ante questionnaire surveyed the following aspects: teachers’ experiences in the school setting as regards food allergies and intolerance; teachers’ level of knowledge. The ex-post questionnaire analysed the following questions: teachers’ level of knowledge; appreciation of the seminar.

A brief presentation of the project was provided in the beginning of each questionnaire, followed by instructions to fill out the latter. Both questionnaires consisted of 36 questions. More specifically, multiple-choice questions were used to assess knowledge levels, while a dichotomous Yes/No format and ten-point Likert rating scales were used in order to evaluate the respondents’ experiences and level of appreciation of the workshop. More details on the questionnaire can be found in Table 2.

**Table 2:** **Structure of questionnaires**

Previous sections of the questionnaire*	Subsequent sections of the questionnaire *
Allergies and intolerance in school: your experience	
In your career as a teacher, have you ever had students with allergies or food intolerance?	
If yes, how may allergic children were you exposed to?	
Considering your experience as a teacher, do you believe that the issue of food allergies is taken into consideration within school environments? Reply with a number from 1 to 10 where 1 = no consideration and 10 = full consideration and attention.	
Have you already participated in courses/seminars for training on food allergies?	
How would you evaluate your level of knowledge on the topic? Reply with a number from 1 to 10, where 1 = very low, 10 = high.	
Allergies and intolerance: knowledge	Allergies and intolerance: knowledge
28 knowledge questions where the respondent had to select an answer deemed correct according to the knowledge and experience of the participants	28 knowledge questions that are the same as those proposed in the previous questionnaire
Personal data:	
Gender	
Age	
	The seminar: your opinion:
	Did the information from the experts provide adequate responses to your informational needs?
	How would you evaluate the relevance of the discussed topics compared to your needs for a training update? Reply with a number from 1 to 10, where 1 = totally irrelevant, 10 = highly relevant
	How would you evaluate the utility of this event for your training/informational updating? Reply with a number from 1 to 10, where 1 = entirely useless, 10 = very useful

*For each question, it was only possible to select one answer from a variety of alternatives.

Descriptive statistics was applied to all variables to analyse the data. In order to compare the teachers’ knowledge level before and after the workshop, the same 28 knowledge questions were used in both questionnaires. In order to assess a potential knowledge increase, two “addition” variables were also constructed by calculating the number of correct answers ex-ante and ex-post respectively. Based on these variables, four groups emerged: “poor knowledge” (number of correct answers ranging between 1 and 7), “fair knowledge” (number of correct answers ranging between 8 and 14), “satisfactory knowledge” (number of correct answers ranging between 15 and 21), and “good knowledge” (number of correct answers ranging between 22 and 28). The Wilcoxon non parametric test for ordinal qualitative variables allowed for an assessment of the significant change in the number of correct answers given ex-ante and ex-post respectively. Data were processed by using the SPSS (Statistical Package for Social Science) software (version 17.0) for Windows (SPSS Inc. Chicago, Illinois).

## Results

### Focus group

The three focus groups involved 25 teachers, all female, mainly aged between 36 and 55 (72%), in line with the gender and age of Italian primary school teachers [35]. It appeared that 15 out of 25 teachers are in charge of the children also during mealtimes in the canteen. The difficulties associated with managing allergies in school and the information needs on the part of teachers were the two main topics which clearly emerged from the focus group analysis.

#### Allergies in school: operational difficulties, competence and responsibility

As regards the management of allergies in school, teachers reported that they are concerned about some practical issues. An emergency becomes difficult to deal with in those cases where a teacher, who is in charge of the class on her own, at the same time has to handle the emergency and keep the other children under control. Moreover, a teacher is not allowed to administer any kind of drug, except in specific cases, and therefore his/her range of action is limited to first aid and to calling for help. In addition, teachers are aware that they do not have sufficient knowledge on this topic. This lack of knowledge causes a feeling of insecurity and inability to correctly manage an emergency situation. Finally, the teachers do not feel prepared to take responsibility for the management of potential allergic reactions, both because they consider it a responsibility which extends beyond their duties and because it involves excessive emotional involvement and worrying. A few comments in this regard are listed in Table 3.

**Table 3:** **Difficulties on the part of teachers in managing allergies in school: a selection of comments made during the focus groups**

**Comments**	
**Operating difficulties in school**	“Emergencies scare us also because we are always face to face with the class … it is very difficult to deal with an emergency because you are on your own” (focus group in Turin)
	“Our hands are somewhat tied: we are often scared but there is not much we can do” (focus group in Rome)
**Teachers’ skills and responsibilities**	“We did receive some training in first aid; however, I am not a doctor and I cannot be forced to do this sort of thing …” (focus group in Rome)
	“Information on a personal level is extremely welcome, of course … when it comes to taking action, it is tough, I am not sure I feel up to it because there is also an emotional element involved; they cannot force me to do something if I don’t feel up to it…” (focus group in Turin)
	“These responsibilities are not part of a teacher’s assignments…” (focus group in Turin)
	“We are not doctors, so I think that these things ought to be done by someone specially appointed, we are still teachers. I cannot do a doctor’s job” (focus group in Rome)

The teachers stressed the importance of involving all children in awareness-raising activities on allergies, not only in order to encourage empathy between allergic children and their schoolmates, but also for the purpose of helping all children become familiar with a problem which could affect them personally.

#### Sources of information

The main sources of direct information appear to be the parents of allergic children. If they have doubts or queries, teachers may also turn to their colleagues, or friends and family members who have experienced similar problems. Generally speaking, teachers feel that there would be a need for a reference such as the school doctor for specific questions and in case of need. Contact with local health units (ASL), as a matter of fact, is limited to serious and certified situations.

With regard to the ways in which they would prefer to receive information about these topics, the teachers, first of all, mentioned direct meetings with experts to whom they could ask questions and receive targeted information. Secondly, the teachers would like to have an information desk available through which they could contact experts in the sector by telephone or through the internet. A further possibility mentioned was the setting up of an online database where they could find practical information and a description of similar case studies.

#### Information needs

In order to most effectively develop the workshops that were planned for the project, the knowledge needs of the teachers were investigated. The topics about which the teachers asked to be informed are:

- difference between allergy and intolerance: understanding the differences both as regards reactions in the body and symptoms;
- indications as to the kind of food which is most at risk: what kinds of food are potentially allergizing;
- indications as to how to recognise an allergic reaction: what can the symptoms be; what can the reaction times be; understanding how serious an allergic reaction may be and its potential development over time;
- basic strategies regarding the management of allergies in school: first aid induction, as well as any instruction in respect of behaviours and actions which could worsen an emergency situation and should thus be avoided.

### Information workshops

The ex-ante and ex-post questionnaires were filled out by 158 teachers, most of them female, aged between 36 and 55 (Table 4).

**Table 4:** **Characteristics of the sample (n = 158)**

**Characteristics**		**%**
Gender	*Male*	4.5
	*Female*	95.5
Age (groups)	*< 35*	10.4
	*36 – 45*	27.9
	*46 – 55*	43.5
	*> 55*	18.2

#### Teachers’ experience

In total, 72.6% of respondents reported having had children suffering from food allergies or intolerance in their teaching experience; in 44.7% of cases one or two of them, in 31.6% of cases between three and five, while in 23.7% of cases more than five.

As for the question “As a teacher, do you consider that food allergies are regarded as a significant issue in school?” (Likert scale: 1 = not significant, 10 = highly significant), the respondents’ mean score was m = 7.6 (SD = 2.1).

Moreover, 27.8% of teachers had already taken part in workshops/training courses on the subject as opposed to 72.2% who had never taken part.

#### Knowledge

Before the seminar started, the teachers were asked for a self-assessment of their level of knowledge on food allergies. In answer to the question “How do you consider your level of knowledge of the subject?” (Likert scale: 1 = very low, 10 = high), the mean score attributed to teachers in relation to their preparation was m = 5.1 (SD = 2.1).

In general, the assessment of ex-ante knowledge showed that, out of 28 knowledge questions, in 16 cases more than 50% of teachers got the answer wrong. The topics where the most serious shortcomings became apparent were first aid, the concept of dose, the development of allergies over time, and milk as a kind of food which may cause allergies or intolerance (Table 5).

**Table 5:** **Subjects and questions in which the teachers made the most mistakes ex-ante**

	**Correct answer**	**Wrong answer%**
**First aid**		
Self-injectable adrenaline should be administered…	Using a syringe, directly through the clothes (e.g. trousers)	84.2
Where should adrenaline ideally be injected?	In the muscle on the side of the thigh	75.9
The adrenaline normally used for emergencies in the case of anaphylactic shock with a self-injecting syringe …	Has a fixed dose	64.6
Is a teacher allowed to administer adrenaline to a child suffering from anaphylactic shock?	Yes, provided that s/he has been duly trained and authorised by the child’s parents	55.1
In case of anaphylactic shock with breathing difficulty and/or dropping of the blood pressure what is the drug of choice which should be used?	Adrenaline	53.8
**Dose and development over time**		
With age food allergies …	May disappear	76.6
If you are hypersensitive to a certain type of food …	It is possible that as you grow older the allergy may disappear	56.3
The dose which triggers a food allergy in an allergic subject …	Depends on the patient	53.2
**Milk-related allergy and intolerance**		
To cure a patient who is intolerant to lactose, the best strategy is …	Avoiding the type of food or ingredient to which s/he is intolerant	63.9
Milk may cause …	Both intolerance and allergy	63.3
Intolerance to milk is due to …	Sugar (lactose) in the milk	60.1

**Figure 1:**
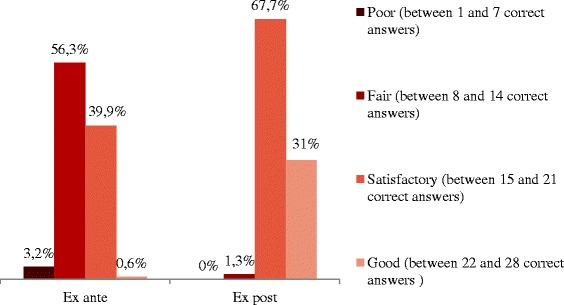
**Percentage of correct answers divided by class, ex-ante and ex-post respectively.**

The knowledge level was assessed by comparing the answers to the 28 multiple-choice knowledge questions included in the ex-ante and ex-post questionnaires. Two “addition” variables were constructed to calculate the number of correct answers given before and after the workshop respectively. On the basis of these variables, four knowledge classes were defined. After the workshop, there was a noticeable shift of the number of correct answers towards the two groups “satisfactory knowledge” and “good knowledge” (Figure 1). More specifically, it emerged that in 72.2% of cases there was a variation in the number of correct answers, while on the other hand in the other 27.8% of cases the results did not change (ZW = −9.77, p = 0.000). This change was not due to neither the age of the respondents (χ2 = 6.188, p = 0.402), nor related to whether they had participated in past training courses (χ2 = 0.143, p = 0.931).

### Assessment of the workshop

As for the questions “Have the presentations by the experts responded appropriately to your information needs?” (available responses: yes, totally; yes, sufficiently; not much; not at all), in total 41.3% of respondents answered yes, very much; 52.9% yes, sufficiently, and 5.8% not much.

As regards the relevance of the topics dealt with compared to the respondents’ training needs (Likert scale 1 = not particularly relevant, 10 = extremely relevant), the mean score amounted to m = 8.4 (±1.66), while the usefulness of the workshop in terms of individual training (Likert scale 1 = not particularly useful, 10 = very useful) showed a mean score amounting to m = 8.6 (1.67).

## Discussion

The data collected for this survey showed a need for communication and training actions geared towards the professional category of teachers. As a matter of fact, even though the latter are in contact with children on a daily basis, they are still insufficiently viewed as a target for communication actions. The research results show that, even though the issue of food allergies is generally perceived as significant, less than one third of the teachers has taken part in specific seminars and courses. This information gap seems to be strongly felt by teachers, who on average do not consider themselves sufficiently prepared on the topic of food allergies and intolerance. This result is in line with that obtained by Ercan in [17]. When asked, in fact, what their reation would be in the case of an anaphylactic reaction on the part of a student, less than 25% of the teachers involved declared that they would provide first aid.

This shortcoming is also confirmed if one considers the teachers’ knowledge level before and after taking part in the workshops. Other studies have revealed insufficient training on the part of school staff members, which could have serious repercussions in terms of managing the problem in school [2,16]. These results emphasize the need for specific educational actions and improvements in school health policies in order to support schools to deal with allergic students and ensure their safety and psychological well-being [30].

An important consequence of such lack of knowledge - and of the perception on the part of teachers that they are not adequately trained - is a feeling of concern and of difficulty in managing possible allergy cases. The need to receive practical information on how to manage allergies, which emerged from the focus groups, was discussed during the workshops. Another important aspect emerging from the focus groups is the importance which teachers attribute to sharing experiences regarding similar cases with friends, family members and colleagues in order to receive information about these problems. The suggestion of a database containing practical instructions and case histories experienced by other teachers confirms the need to discuss and share their experiences, even from an emotional viewpoint.

The teachers, moreover, underscored the importance of the expert’s role in order to have reliable information on the subject. Training workshops - during which it should be possible to discuss any doubts and specific questions with specialists in the sector - seem to be, in the teachers’ opinion, an effective method to acquire information. The workshops organized in the project, in fact, were highly appreciated by participants who confirmed their usefulness. The wish expressed by teachers to talk to experts goes beyond the specific occasion offered by a meeting. According to the teachers, it would be important to have a point of reference for health issues such as food allergies. Training and education on this topic, indeed, need to be continuous and require on-going updates, even in relation to specific allergy cases in a school and their development over time [36]. Numerous studies, also at international level, have addressed the importance of a constant relationship between families, school staff and medical staff, in order to make sure that the problem is managed with appropriate collaboration [22,37]. Communication between these various figures is a relevant aspect which would also need greater consideration, even within the Italian school setting.

During the focus groups with teachers, the centrality of the child was highlighted, not only in relation to an allergic child but in relation to all children. The interest on the part of teachers in awareness-raising actions and training projects aimed at involving children is a very clear indication which ought to be taken into account.

## Conclusions

This study has surveyed the information needs of a group of primary school teachers in relation to food allergies; based on the collected information, training actions have been designed, implemented and monitored. The data analysis revealed some knowledge gaps on the part of teachers, and a lack of training action in this respect. These results underline the urgency of training actions specifically devoted to allergies in the school setting, aimed in particular at raising awareness among teachers, disseminating practical information for the management of allergy cases and emergencies as well as at clarifying the levels of responsibility involved in medical actions in a non-healthcare environment. The intervention study presented here showed some limitations due to its experimental nature on a limited sample; as a result, it is not possible to draw general conclusions from the results obtained for primary school teachers as a whole. However, the project development in its various phases may provide useful indications for an in-depth analysis of the topic in the Italian setting, as well as for implementing ad-hoc communications actions geared towards teachers, children and all families.
